# Identification of Genes Involved in the Differentiation of R7y and R7p Photoreceptor Cells in *Drosophila*

**DOI:** 10.1534/g3.120.401370

**Published:** 2020-09-24

**Authors:** James B. Earl, Lauren A. Vanderlinden, Thomas L. Jacobsen, John C. Aldrich, Laura M. Saba, Steven G. Britt

**Affiliations:** * Department of Cell and Developmental Biology, School of Medicine, University of Colorado Anschutz Medical Campus, Aurora, CO, 80045; † Department of Biostatistics and Informatics, Colorado School of Public Health, Aurora, CO 80045; ‡ Department of Neurology, Department of Ophthalmology, Dell Medical School, University of Texas at Austin, Austin, TX 78712; § Department of Pharmaceutical Sciences, Skaggs School of Pharmacy and Pharmaceutical Sciences, University of Colorado Anschutz Medical Campus, Aurora, CO 80045

**Keywords:** Photoreceptor, Cell fate, Gene inactivation, Ectopic expression, Mutant screen

## Abstract

The R7 and R8 photoreceptor cells of the *Drosophila* compound eye mediate color vision. Throughout the majority of the eye, these cells occur in two principal types of ommatidia. Approximately 35% of ommatidia are of the pale type and express *Rh3* in R7 cells and *Rh5* in R8 cells. The remaining 65% are of the yellow type and express *Rh4* in R7 cells and *Rh6* in R8 cells. The specification of an R8 cell in a pale or yellow ommatidium depends on the fate of the adjacent R7 cell. However, pale and yellow R7 cells are specified by a stochastic process that requires the genes *spineless*, *tango* and *klumpfuss*. To identify additional genes involved in this process we performed genetic screens using a collection of 480 *P{EP}* transposon insertion strains. We identified genes in gain of function and loss of function screens that significantly altered the percentage of *Rh3* expressing R7 cells (*Rh3*%) from wild-type. 36 strains resulted in altered *Rh3*% in the gain of function screen where the *P{EP}* insertion strains were crossed to a *sevEP-GAL4* driver line. 53 strains resulted in altered *Rh3*% in the heterozygous loss of function screen. 4 strains showed effects that differed between the two screens, suggesting that the effect found in the gain of function screen was either larger than, or potentially masked by, the *P{EP}* insertion alone. Analyses of homozygotes validated many of the candidates identified. These results suggest that R7 cell fate specification is sensitive to perturbations in mRNA transcription, splicing and localization, growth inhibition, post-translational protein modification, cleavage and secretion, *hedgehog* signaling, ubiquitin protease activity, GTPase activation, actin and cytoskeletal regulation, and Ser/Thr kinase activity, among other diverse signaling and cell biological processes.

Color vision in most organisms is dependent upon the expression of spectrally distinct visual pigments (opsins) in different photoreceptor cells (Jacobs 1981; Nathans *et al.* 1986; Wikler and Rakic 1990) . The organization of the retinal mosaic reflects a variety of developmental mechanisms, including regional specialization, stochastic, and precise cell-cell adjacency (Viets *et al.* 2016). *Drosophila melanogaster* is capable of color vision and is a useful experimental system for examining the developmental programs that produce photoreceptor cells having different color sensitivities (Quinn *et al.* 1974; Spatz *et al.* 1974; Chou *et al.* 1996; Chou *et al.* 1999; Tang and Guo 2001; Cook *et al.* 2003; Wernet *et al.* 2003; Mikeladze-Dvali *et al.* 2005). The compound eye consists of ∼800 ommatidia, which each contain a pair of R7 and R8 photoreceptors cells that mediate polarization sensitivity and color vision (Fortini and Rubin 1991; Yamaguchi *et al.* 2010).

Two main ommatidial subtypes were identified based on pale or yellow fluorescence when illuminated with blue light (Kirschfeld *et al.* 1978; Franceschini *et al.* 1981), and contain R7pale/R8pale (R7p/R8p) or R7yellow/R8yellow (R7y/R8y) cell pairs expressing *Rh3*/*Rh5* or *Rh4*/*Rh6*, respectively (Chou *et al.* 1996; Papatsenko *et al.* 1997; Chou *et al.* 1999). The R7y and R7p photoreceptor cells are distributed randomly (Bell *et al.* 2007) and thought to be generated stochastically through the action of *spineless** (ss)*, *tango* (*tgo*) and *klumpfuss** (klu)* (Wernet *et al.* 2006; Thanawala *et al.* 2013; Johnston and Desplan 2014; Anderson *et al.* 2017). The premise behind this stochastic cell-fate mechanism is that variation in the expression of *ss* in individual R7 cells in the developing pupal eye leads to the formation of R7y and R7p cells. This is consistent with the idea that regulators of this process may be rate-limiting or define dosage-sensitive steps in the formation of R7y cells. Cell to cell variation in gene expression may arise as the result of promoter architecture (Jones *et al.* 2014), transcriptional or translational bursting (Mcadams and Arkin 1997; Singh and Soltani 2013), cell-cell interactions (Axelrod 2010), epigenetic differences between cells (Kar *et al.* 2017) and other processes. To identify additional regulators of R7 photoreceptor cell differentiation, we conducted Gain of Function (GOF) and Loss of Function (LOF) genetic screens to identify genes that govern this process. Here we show that mutations in many additional genes regulate the proportion of R7y and R7p cells.

## Methods & Materials

### Drosophila stocks and genetics

All stocks were maintained in humidified incubators on standard cornmeal/molasses/agar media at 25°. Stocks used in the experiments described here were obtained from the Bloomington *Drosophila* Stock Center and included *w^1118^* (FBst0003605) and *w^1118^*; *P{w+mW.hs = sevEP-GAL4.B}7* (FBst0005793) (*w*; *sevEP-GAL4*). The *sevEP-GAL4* strain was selected because it is expressed at a higher level and is more cell type specific than *P{GAL4-Hsp70.sev}* (FBtp0000379), which is driven by the Hsp70 promoter and is thought to have *sevenless* independent activity (Bailey 1999; Therrien *et al.* 1999). The 480 *P{EP}* (FBtp0001317) (Rorth 1996) transposon insertion strains used in the screen are listed in Supplemental Table S1. Additional stocks used in Table 2 include w*; *ss*^*D115.7*^* / TM3*, *P{ry^+t7.2^ = HB-lacZ}GS2*, *Sb*^*1*^ (FBst0078357) and w*; *P{w^+mC^ = UAS-ss.A5}A1 / SM6a* (FBst0078354).

### Screen Design

The goal of this project was to identify genes that alter the percentage of *Rh3* expressing R7 cells (*Rh3*%). This was undertaken in two screens. First, a gain of function (GOF) screen was performed by crossing each *P{EP}* strain to *w*; *sevEP-GAL4* flies. Second, a heterozygous loss of function (LOF) screen was performed by crossing each *P{EP}* strain to *w^1118^* flies. For each cross 8-10 female F1 progeny were used to determine the number of R7 photoreceptor cells expressing *Rh3* and *Rh4* (*Rh3*%). In an additional analysis, we calculated the difference (Diff) between the two screens (Diff = GOF – LOF) for each *P{EP}* insertion strain. This was designed to identify effects found in the GOF screen that are larger than, or potentially masked by, the effect from the *P{EP}* insertion alone, as observed in the heterozygous LOF screen.

### Description of phenotypes scored

Immunofluorescence of R7 photoreceptor cells was performed on dissociated ommatidia, prepared as described (Hardie *et al.* 1991; Ranganathan *et al.* 1991). *Rh3* and *Rh4* were detected by immunohistochemistry using mouse monoclonal antibodies as previously described (Chou *et al.* 1996; Chou *et al.* 1999). Antibodies were diluted in antibody dilution buffer (3% normal goat serum, 0.03% triton X-100, and 0.1% bovine serum albumin in PBS). Dilutions used for indirect immunofluorescence were as follows: 1:20 anti-*Rh3* mouse monoclonal (clone 2B1, IgG1); 1:10 anti-*Rh4* mouse monoclonal (clone 11E6, IgG1). For direct immunofluorescence, directly conjugated anti-*Rh3*-Alexa Fluor 488 was used at 1:50 (Earl and Britt 2006). Secondary antibodies and other immunological reagents were obtained from Jackson ImmunoResearch Laboratories, Inc. and Molecular Probes. Images were collected using an Axioskop2 Plus / AxioCamHRC microscope (Carl Zeiss, Inc.; Thornwood, NY).

### Statistical analyses

Dissociated ommatidia were examined by fluorescence microscopy and the number of R7 cells expressing *Rh3* or *Rh4* were counted in each sample. To account for varying number of R7 cells examined for each cross, logistic regression with *Rh3* expression (yes/no) as the outcome at the individual R7 cell-level was used to examine the association between a specific mutant in the GOF screen and the probability of a cell expressing *Rh3*. An odds ratio was constructed to compare the odds of a cell expressing *Rh3* in a specific mutant to the odds of a cell expressing *Rh3* in all other mutants from the same chromosome. We adjusted for multiple testing by applying a False Discovery Rate (FDR) correction across all comparisons (all mutant strains from all chromosomes) (Benjamini and Hochberg 1995). An FDR threshold of 0.10 was used to identify mutant strains associated with the differences in the probability of an R7 cell expressing *Rh3*. The same model was used to determine if the probability of *Rh3* expression for a specific mutant strain is significantly altered in the LOF screen. Effect size is reported as a standardized percent *Rh3*, which is the difference between the mutant strain specific percent *Rh3* and the median percent *Rh3* for all mutant strains on that chromosome. Cells with inconclusive calls were not considered in the analysis.

We also examined whether the *difference* in the probability of *Rh3* expression between the GOF screen and the LOF screen for a specific mutant strain was significantly lower/higher than other mutant strains on the same chromosome. A logistic regression model for each mutant strain was estimated using an indicator for the specific mutant strain being tested (yes/no), type of mutation (GOF or LOF) and the interaction effect between these two factors. The interaction effect from this model was used to determine if the effect of a particular *P{EP}* allele differed between the two screens (GOF or LOF). An FDR correction was used for multiple testing and a threshold of 0.10 was used to identify candidate genes.

### Statistical analysis of specific genotypes

Comparisons of the proportions (percentages) of opsin expression in different genetic backgrounds were performed with a *z*-score and are shown in Table 2 (Fleiss *et al.* 2003). The *z*-score was calculated using the equation:

z=[ρ2−ρ1]−12(1n1+1n2)ρavgqavg(1n1+1n2)

*p_1_* and *p_2_* = proportions of marker expression in each of the two different genotypes under comparison. *n_1_* and *n_2_* = number of ommatidia counted for each genotype. *p_avg_* = average proportion for both genotypes combined. *q_avg_ =* 1-*p_avg_*. The significance of the difference between the two proportions was determined from the normal distribution as a two-tailed test.

### Network analysis

esyN (version 2.0) (Bean *et al.* 2014) was used to construct networks involved in R7 cell fate by extracting known genetic and physical interactions among candidate genes from the mutant screens and *tgo*, *ss** and **klu*, genes known to be involved in R7 cell fate specification (based on FlyBase, version FB2018_04). Networks were restricted to include only connections that involved at least one candidate gene.

### Validating candidate genes

To validate selected candidates, we tested *P{EP}* strains as homozygotes as described above. A standardized *Rh3*% was calculated for the Homozygous Loss of Function (HomLOF) data and compared to the median *Rh3*% for each chromosome from the Heterozygous Loss of Function (HetLOF) screen.

### Data availability

The data described in this manuscript is available as Supplemental Tables S1, S2 and S3 available at figshare: https://doi.org/10.25387/g3.12994292

## Results

### Screen design

**Figure 1:**
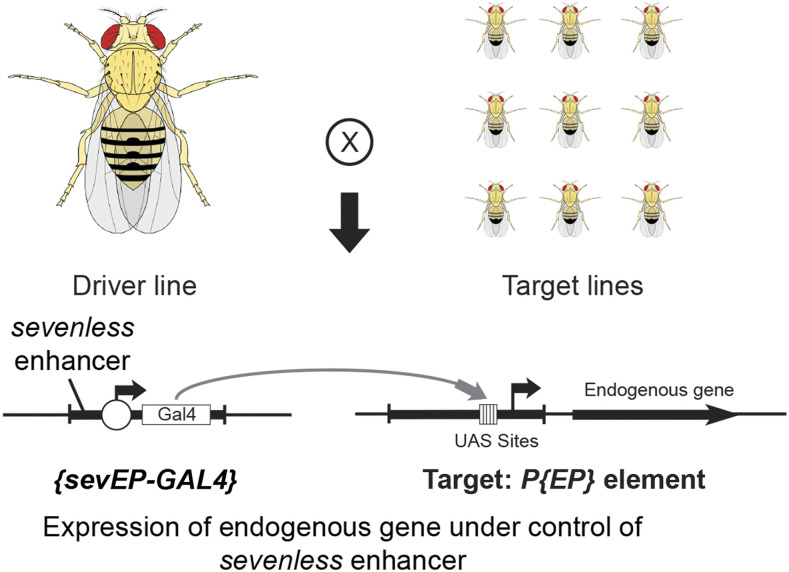
Overview of the *P{EP}* Transposon Insertion GAL4/UAS System and its use in the Gain of Function Screen. The genetics underlying the Gain of Function (GOF) Screen includes a Driver line (left) and Target lines (right). The Driver line is a transgenic strain that carries a transposon insertion *{sevEP-GAL4}* containing a DNA construct composed of the *sevenless* enhancer, a minimal promoter, transcription start site and the coding region of the yeast GAL4 transcription factor. In this strain, GAL4 is expressed in the pattern of *sevenless* protein and is produced dynamically in the 3^rd^ instar larval eye-antenna imaginal disc, posterior to the morphogenetic furrow in R7 photoreceptor and other cells (Tomlinson *et al.* 1987; St Pierre *et al.* 2002; Ray and Lakhotia 2015). The Target lines are transgenic strains that carry independent insertions of the *P{EP}* transposon at different locations in the fly genome. The *P{EP}* transposon contains a series of yeast Upstream Activating Sequence sites (UAS sites, vertical bars in a block) that are the target of GAL4 binding. Upon mating of a Driver line carrying *{sevEP-GAL4}* to a single Target line carrying a unique *P{EP}* insertion, the offspring will express GAL4 in R7 photoreceptors. GAL4 will bind to and activate transcription from UAS sites of the adjacent endogenous gene or genomic region. In this manner, an endogenous gene may be expressed under the control of the *sevenless* enhancer. Figure modified from (Rorth *et al.* 1998). *Drosophila* illustration modified from (Commons 2017).

**Figure 2:**
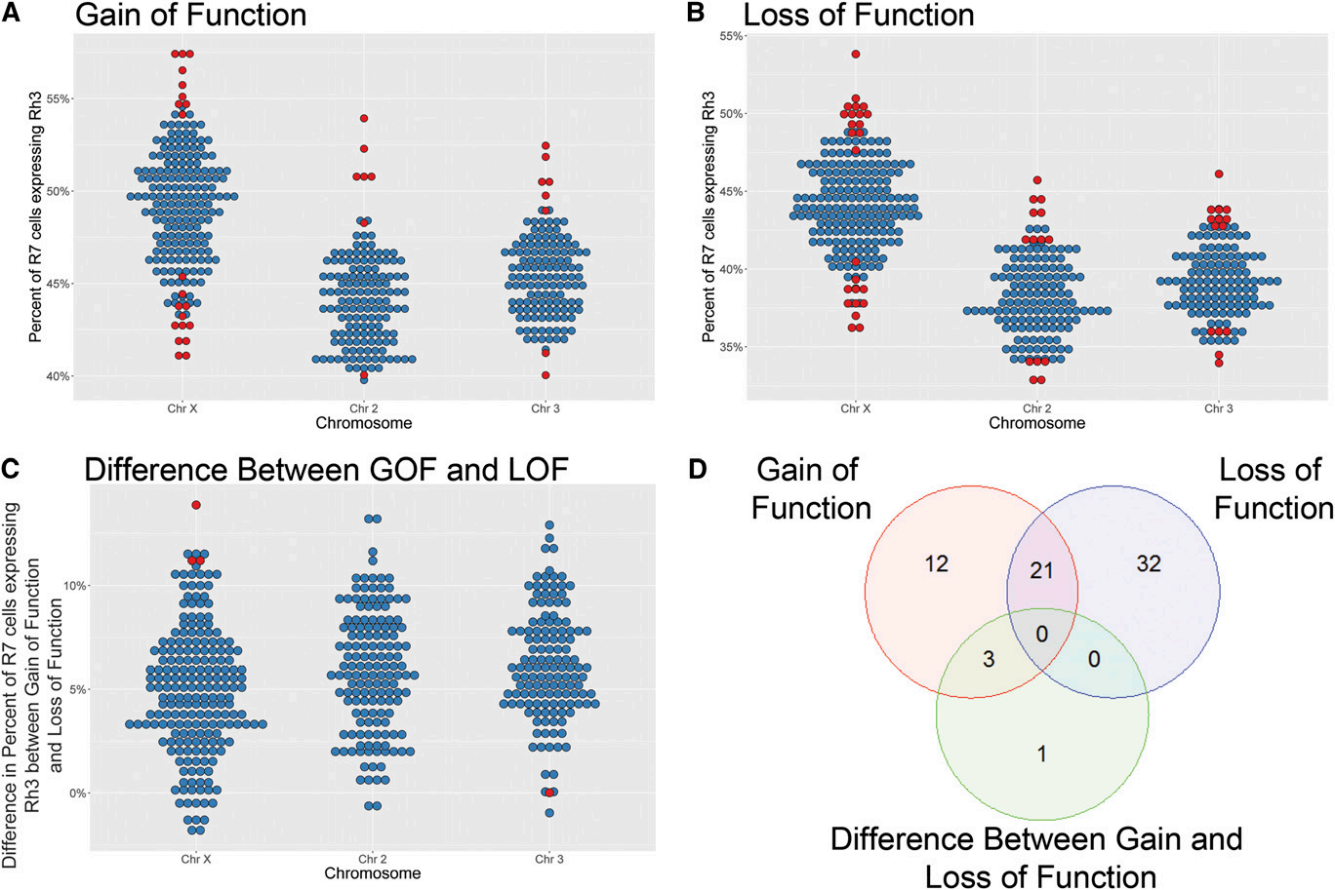
Percent of R7 cells expressing *Rh3* in P-element insertion screens. A) The panel shows the *Rh3*% measured for animals from the Gain of Function (GOF) screen in which individual *P{EP}* strains were crossed to *sevEP-GAL4*. The panel is organized by the chromosome of the insertion. Individual strains were compared to the median *Rh3*% for strains on that chromosome. Dots indicate individual insertion strains. Red dots correspond to measurements where the *Rh3*% was significantly lower / higher with a False Discovery Rate (FDR) < 0.10. Blue dots correspond to all other insertions. B) The panel shows the *Rh3*% measured for animals from the heterozygous Loss of Function (LOF) screen, which carry a single copy of the *P{EP}* transposon at a specific genomic location. The data are presented as in Panel A. C) The panel shows the Difference (Diff) comparison between the GOF and LOF screens. The y-axis represents the difference in the *Rh3*% between the two screens (Diff = GOF - LOF) and was designed to identify effects found in the GOF screen that are larger than, or potentially masked by, the effect of the *P{EP}* insertion alone, as observed in the heterozygous LOF screen. The data are presented as in Panel A. D) The panel shows the overlap of candidate genes among the GOF and LOF screens and the Diff comparison. Each circle represents a different test. Twenty-one candidates are shared between GOF and LOF screens. Three candidates are shared between the GOF screen and Diff comparison. The remaining candidates were identified in a single screen or the Diff comparison.

The genetic screens described here were developed to identify new genes involved in the cell fate decision that regulates *Rh3* *vs.* *Rh4* expression in R7p and R7y photoreceptors cells, respectively. Three genes, *ss*, *tgo* and *klu* are required for this process (Wernet *et al.* 2006; Thanawala *et al.* 2013; Johnston and Desplan 2014; Anderson *et al.* 2017) and we have shown that the cell fate decision between R7p and R7y also depends upon the *Epidermal growth factor receptor* (*Egfr*) (Birkholz *et al.* 2009a). We used the *P{EP}* transposable element (Rorth 1996), taking advantage of its utility 1) as a modular GAL4 system for over- or mis-expression in a Gain of Function (GOF) Screen (Figure 1) and 2) as a single *P*-element mutagenesis experiment performed as a heterozygous Loss of Function (LOF) Screen. The rationale for these screens is that the stochastic expression of *ss* and the specification of R7y or R7p photoreceptor cells may be dependent upon the precise expression level of a regulator that is rate-limiting for this process. Such a regulator could potentially be identified by its effect on R7 photoreceptor cell differentiation when the concentration, expression or gene dosage of the regulator is altered within the system.

Our approach using both a traditional GOF and a heterozygous LOF screen is based on the observation that *ss* is required for expression of *Rh4* in R7 cells. Homozygous *ss* mutant patches show a dramatic decrease in *Rh4* expressing R7 cells and a corresponding increase in *Rh3* expressing R7 cells (Wernet *et al.* 2006). In addition, overexpression of *ss* leads to expression of *Rh4* in most or all R7 cells (Wernet *et al.* 2006; Johnston and Desplan 2014). Finally, *ss* mutants show a dosage effect, in that loss of a single copy of *ss* leads to an increase in *Rh3* expressing R7 cells from 35 to 44%, an increase of 9% from wild-type controls (Johnston and Desplan 2014). These findings suggest that genetic screens designed to increase or decrease expression of rate limiting regulators of *Rh3* and *Rh4* expression may identify additional genes involved in this process.

### Screen results

In the GOF screen, each *P{EP}* target line was crossed to the *w*; *sevEP-GAL4* driver line (Figure 1). In this strain, GAL4 is expressed in the pattern of *sevenless* protein and is produced dynamically in the 3^rd^ instar larval eye-antenna imaginal disc, posterior to the morphogenetic furrow in photoreceptor cells R3, R4, the mystery cells, R1, R6, R7, the cone cells, and some additional cells in the Central Nervous System (Tomlinson *et al.* 1987; St Pierre *et al.* 2002; Ray and Lakhotia 2015). An average of over 700 ommatidia were counted for each cross (range: 179-2312). The median of *Rh3*% for each chromosome of the GOF screen are 49.5%, 44.0% and 45.3% for chromosomes X, 2 and 3 respectively, Figure 2A.

In the heterozygous LOF screen, each *P{EP}* strain was crossed to *w^1118^*. The median of *Rh3*% for each chromosome of the LOF screen are 44.0%, 38.0% and 39.1% for chromosomes X, 2 and 3 respectively, Figure 2B.

We also examined whether the *difference* in *Rh3*% between the GOF screen and the LOF screen for a specific mutant strain was significantly lower/higher than other mutant strains on the same chromosome. This was designed to identify effects found in the GOF screen that are larger than, or potentially masked by, the effect of the *P{EP}* insertion alone, as observed in the heterozygous LOF screen. We calculated the difference (Diff) between the two screens (Diff = GOF – LOF) for each *P{EP}* insertion strain. The median of *Rh3*% Diff for each chromosome are 5.0%, 5.9% and 5.9% for chromosomes X, 2 and 3 respectively, Figure 2C. The medians for *Rh3*% for different chromosomes in the GOF and LOF screens, and the Diff comparison, likely vary because of the genetics and varying sources of the chromosomes used to generate the hopped *P{EP}* strains. The median *Rh3*% of the Diff comparison is non-zero and we believe that this results from an effect of the strains carrying a copy of only the *P{EP}* transposon in the LOF screen *vs.* carrying a copy of both the *P{EP}* and *sevEP-GAL4* transposons in the GOF screen. Performing a non-parametric Kruskal-Wallis test for differences between group medians, there are significant differences across chromosomes in the median percentage of cells expressing *Rh3* in the GOF and LOF screens and Diff comparison (*p*-values 6.3 × 10^−41^, 1.9 × 10^−52^ and 6.7 × 10^−4^, respectively).

Based on the genomic site of the *P{EP}* insertion, we identified 36 candidate genes in the GOF screen (Figure 2A; Supplemental Table S2), 53 in the LOF screen (Figure 2B; Supplemental Table S2) and 4 from the Diff comparison (Figure 2C; Supplemental Table S2) having an FDR < 0.10. Some genes appeared in multiple screens resulting in 69 unique candidate genes in total (Figure 2D). Of the 36 candidate genes from the GOF screen, 21 (58.3%) exhibited an increase in *Rh3*% compared to other GOF mutants from the same chromosome. Likewise, of the 53 candidate genes from the LOF screen, 32 (60.4%) showed an increase in *Rh3*% compared to other LOF mutants on the same chromosome. Of the 4 mutants identified in the Diff comparison, 3 (75%) indicated a difference in *Rh3*% levels between GOF and LOF screens that were greater than the Diff median for that chromosome.

### Top candidates

For a more qualitative examination, we prioritized 10 of the 69 candidate genes (Table 1). These 10 include the 4 candidate genes from the Diff comparison and the top 3 candidates based on *p*-value from the GOF and LOF screens. In 5 of the 10 candidate genes, the LOF mutant did not result in a significant change in *Rh3*%, but the GOF screen did. In 4 of these 5, (*IGF-II mRNA-binding protein* (*Imp*), *grauzone* (*grau*), *Tyrosylprotein sulfotransferase* (*Tpst*), and *Furin 2* (*Fur2*)), *Rh3*% increased with GOF. GOF of the other gene, *Small ribonucleoprotein particle protein SmD2* (*SmD2*), led to a decrease in *Rh3*%. In 5 of the remaining mutant strains, two (*Tao* and *Phospholipase A2 group III* *(**GIIIspla2*)) had a significant decrease in *Rh3*% in the LOF mutants, but the magnitude of the decrease did not change significantly with GOF. Both *Actin-related protein 3* (*Arp3*) and *Rho GTPase activating protein at 18B* (*RhoGAP18B*) had a significant increase in *Rh3*% in their respective LOF mutants, but for both mutations, that increase was dampened with GOF. For both genes, the GOF screen and the Diff comparison were suggestive (unadjusted p-values between 0.03 and 0.11) of a difference, but none of the four comparisons reached statistical significance after multiple testing correction. For the final gene in Table 1, G *protein β-subunit 13F* (*Gβ13F*), LOF causes a slight up-regulation in *Rh3*%, while GOF caused a slight down-regulation in *Rh3*%. Both comparisons were nominally significant but failed to pass multiple testing correction, but the Diff comparison was statistically significant.

**Table 1:** Selected Mutants with Significant Differences in R7 cells expressing *Rh3*

Allele	Chr	Screen with FDR < 0.10	Gain of Function *Rh3*% (GOF)	Loss of Function *Rh3*% (LOF)	Difference (Diff)	EP Orientation	EP Position
*SmD2^EP3399^*	3	GOF & Diff	−5.29% (2.25E-05)	0.94%	−5.89%	forward	5′ end
*Tpst^EP1218^*	X	GOF & Diff	7.80% (8.76E-11)	2.10%	6.11%	forward	5′ end
*Fur2^EP1493^*	X	GOF & Diff	7.06% (3.96E-07)	−1.39%	8.87%	reverse	5′ end
*Gβ13F^EP1071^*	X	Diff	2.54%	−3.31%	6.27% (5.75E-04)	reverse	5′end
*grau^EP688^*	2	GOF	6.94% (2.14E-06)	2.94%	4.18%	forward	5′end
*Imp^EP760^*	X	GOF	8.10% (1.42E-07)	2.59%	5.93%	forward	1^st^ intron
*Arp3^EP3640^*	3	LOF	2.87%	7.03% (1.82E-05)	−3.82%	forward	5′ end
*RhoGAP18B^EP1326^*	X	LOF	3.11%	6.93% (1.35E-05)	−3.40%	reverse	mid or 1^st^ intron
*Tao^EP1455^*	X	GOF & LOF	−8.20 (2.17E-09)	−6.08%	−1.70%	forward	5′ end
*GIIIspla2^EP1516^*	X	GOF & LOF	−8.54%	−7.63% (1.35E-08)	−0.49%	reverse	5′end

The Table shows the top candidate alleles identified in the study, the Chromosome (Chr) containing the *P{EP}* insertion and the screens for these alleles showing significant effects. Abbreviations: False Discovery Rate (FDR), Standardized Percent of *Rh3* expressing R7 cells (*Rh3*%), Gain of Function (GOF), heterozygous Loss of Function (LOF), Difference between primary screens (Diff) = GOF *Rh3*% - LOF *Rh3*%. The table includes the top 3 candidates from the GOF and LOF screens, and 4 candidates showing a significant Diff comparison. EP Orientation refers to the direction of transcription from the *P{EP}* insertion relative to the indicated gene. EP Position refers to the position of the *P{EP}* insertion relative to the transcript of the indicated gene. The Standardized *Rh3*% refers to the difference between the mutant specific *Rh3*% and the median *Rh3*% across all mutants on the same chromosome for that screen. Shaded cells indicate measurements that meet an FDR < 0.10 and the *p*-value of the highest statistical significance for that mutant strain is also shown.

### Comparison of GOF and LOF screen results with analyses of spineless

As an internal reference for the GOF screen, we expressed *ss* under the control of the *sevEP-GAL4* driver line (Strain # 4, Table 2). Previous studies demonstrated that overexpression of *ss* under the control of the *P{longGMR-GAL4}* or the *P{GAL4-ninaE.GMR}* driver lines leads to expression of *Rh4* in almost all R7 and many R1-6 photoreceptor cells (Wernet *et al.* 2006). By contrast, our experiments with the more restricted *sevEP-GAL4* driver line showed no statistically significant change in Rh3 and Rh4 expression in R7 cells compared to the *P{UAS-ss}* responder line alone (Strain # 3, Table 2). This discrepancy likely reflects a difference in the location and/or timing of GAL4 expression in the driver lines. Despite the failure of *sevEP-GAL4* driven *ss* expression to alter Rh3% in the screening paradigm, 36 statistically significant candidates were identified in the GOF screen.

**Table 2:** R7 Photoreceptor Opsin Expression in Different Genetic Backgrounds

Strain #	Genotype Brief description	*Rh3%* (n)	Statistical Comparison
1	*w^1118^*** Control**	35.5 (363)	
2	*w^1118^*; *P{w^+mW.hs^ = sevEP-GAL4.B}7 / +* ***{sevEP-GAL4}* driver line**	35.1 (427)	NSDF #1
3	w^1118^ / w*; *P{w^+mC^ = UAS-ss.A5}A1 / +* ***{UAS-ss}* responder line**	45.0 (329)	SDF #1 *P* = 0.01
4	w^1118^/ w*; *P{w^+mW.hs^ = sevEP-GAL4.B}7 / P{w^+mC^ = UAS-ss.A5}A1* ***{sevEP-GAL4}* driving *{UAS-ss} expression***	51.9 (391)	NSDF #3
5	*w^1118^ / Y* (♂) **Control**	30.6 (214)	NSDF #1
6	*w^1118^ / Y*; *ss^D115.7^ / +* (♂) ***ss* heterozygote**	36.2 (472)	NSDF #5

Statistical comparisons of strains were carried out as described in the Methods; Percent of *Rh3* expressing R7 cells (*Rh3*%), n= the number of ommatidia counted. As indicated, the observed percentages were Not Significantly Different From (NSDF, *P* ≥ 0.05) or Significantly Different From (SDF) the strain indicated, at the *p* value shown by a two tailed test. Tested animals were female unless otherwise indicated.

As mentioned in the introduction, the rationale for the heterozygous LOF screen is the prediction that loss of a single copy of a rate limiting or dosage sensitive regulator of R7 cell differentiation would alter Rh3%. Loss of a single copy of *ss* has been shown to increase the proportion of R7 cells that express *Rh3* from 35 to 44% (9% change) (Johnston and Desplan 2014). This effect, observed in *Df(3R)Exel7330* heterozygotes, is comparable to the maximum effect we observed for heterozygous *P{EP}* insertions in the LOF screen (Table 1, Figure 2B). However, our comparison of heterozygous *ss*^*D115.7*^ (Strain # 6) animals to *w^1118^* (Strain # 5) controls showed a 5.6% increase in Rh3%, that is not statistically significant (Table 2). The discrepancy between the two results may be due to the use of differing alleles or genetic backgrounds. *Df(3R)Exel7330* contains a 125Kb deletion removing approximately 20 genes (FBab0038318), whereas *ss*^*D115.7*^ is a 10Kb intragenic deletion within *ss* (Duncan *et al.* 1998). Furthermore, the previous study did not include specific experimental values sufficient for statistical comparison with relevant controls, or with our results. Thus, the 53 statistically significant candidates identified in the heterozygous LOF screen exceed expectations.

### Potential interactions between candidates and known regulators

As a first step in defining the relationship between the 69 candidate genes identified in the screen and the well characterized mediators of R7 photoreceptor cell differentiation and the regulation of *Rh3* *vs.* *Rh4* expression, *ss*, *tgo* and *klu*, we examined the known genetic and physical interactions between these genes. Of the 69 candidate genes, 66 were included in the esyN database. 38 candidate genes have known interactions with another candidate(s) or an interaction with another gene 1 link away from another candidate(s).

**Figure 3:**
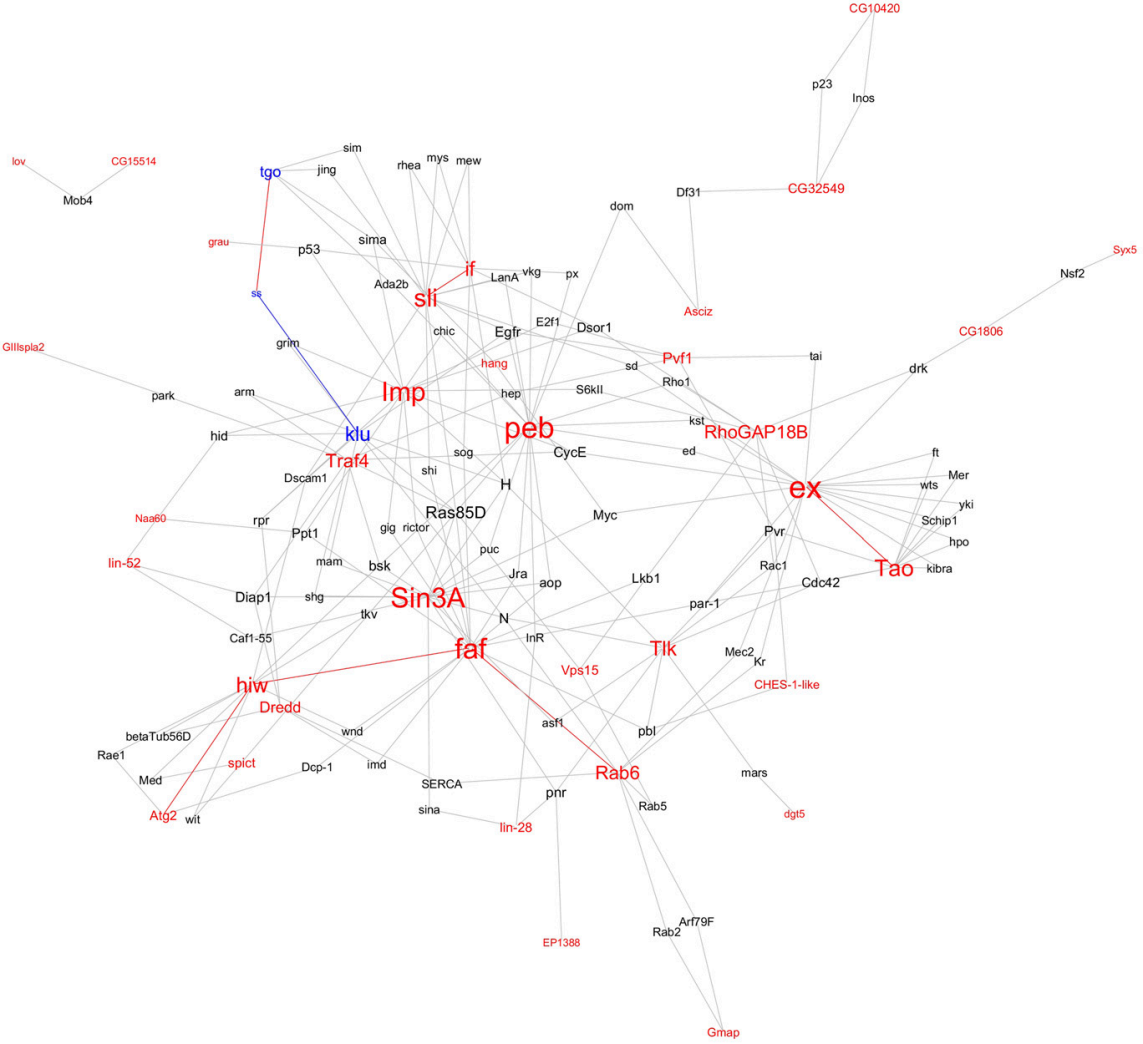
Interaction network associated with R7 cell fate differentiation and the expression of *Rh3* and *Rh4*. Candidate genes identified in the mutant screens that alter the percent of R7 cells expressing *Rh3* are shown in red. *spineless** (ss)*, *tango** (**tgo**)* and *klumpfuss (klu)*, which are known from previous studies to regulate *Rh3*%, are shown in blue. Genes with a known genetic or physical interaction with these genes are shown in black. Direct links between candidate genes are shown in red. The link between *klu* and *ss* is labeled blue (Anderson *et al.* 2017). The font size of gene name is based on the number of other genes it is connected to. Supplemental Table S3 lists the genes in the network and the total number of connections for each gene.

This analysis identified 85 additional genes that interact physically or genetically with the candidates identified in the screen and *ss*, *tgo* or *klu*. These additional genes have known interaction with two or more candidate genes (Figure 3). Most candidate genes are in a single large network where the genes *expanded* (*ex*) and *pebbled* (*peb*) were the most highly connected (*i.e.*, hub genes). This large network contains the known regulators of R7 photoreceptor cell specification *ss*, *tgo* and *klu*. Although *ss*, *tgo* and *klu* do not have known direct genetic or protein interactions with any of the candidate genes identified in the screen, there are numerous secondary interactions with candidate genes throughout the network, including *peb*, *Imp*, *inflated* (*if*), *slit* (*sli*), *TNF-receptor-associated factor 4* (*Traf4*) and others. A much smaller network consists of candidate genes *CG15514* and *jim lovell* (*lov*) connected by *MOB kinase activator 4* (*Mob4*). Only a single link is shown between a pair of genes even if multiple types of connections have been identified (*e.g.*, a suppressing genetic interaction and a physical interaction). The 85 additional connecting genes in the network were not evaluated in the mutant screen, except for *transcriptional Adaptor 2b* (*Ada2b*), and *scalloped* (*sd*) (insertions *P{EP}EP3412* and *P{EP}sd*^*EP1088*^, respectively), which did not reach statistical significance. Eight candidate genes (*ex*, *peb*, *fat facets* *(**faf**)*, *Sin3A*, *Imp*, *sli*, *Tao* and *highwire* (*hiw**)*) are highly embedded within the network and have connections to 10 or more other genes. These interaction networks suggest numerous mechanisms for regulation of *ss*, *tgo* and *klu* by the genes identified in the screens. Supplemental Table S3 lists the genes in the network and the total number of connections for each gene.

### Candidate gene validation

**Figure 4:**
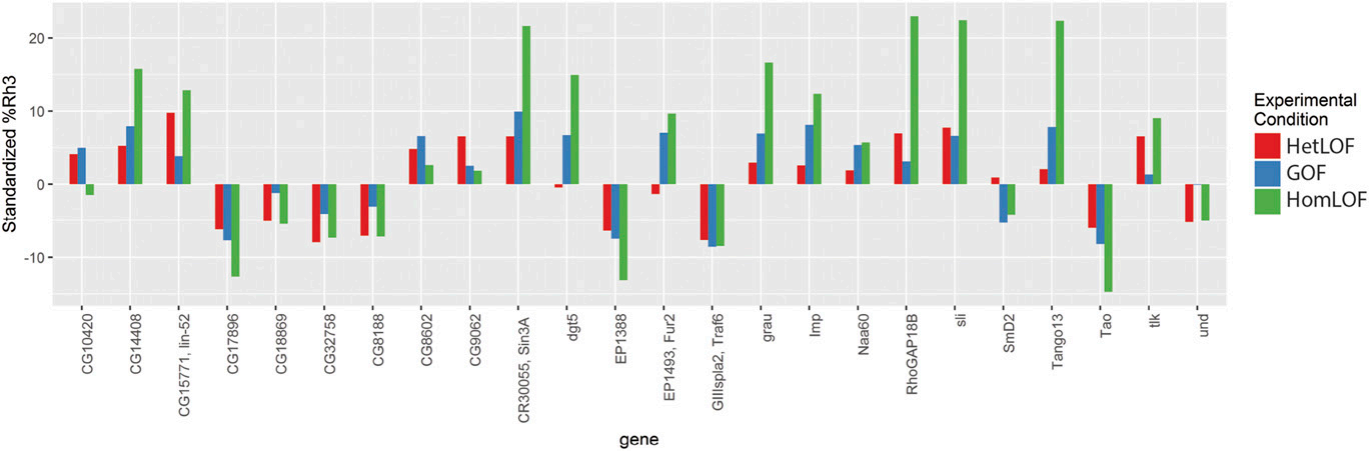
Homozygous Loss of Function validation of selected candidates from mutant screens. The bar graph shows the standardized *Rh3*% for animals in the Heterozygous Loss of Function (HetLOF, red bars) and Gain of Function (GOF, blue bars) screens. *Rh3*% for animals in the Homozygous Loss of Function (HomLOF) validation are shown in green. *Rh3*% of HomLOF were examined for 28 selected candidates. Four of these mutants (*Arp3*, *Autophagy-related 2,* (*Atg2*), *faf*, and *lov*) were homozygous lethal and are not included in the figure. A standardized *Rh3*% was calculated for the HomLOF data using the *Rh3*% median for each chromosome from the HetLOF screen.

**Figure 5:**
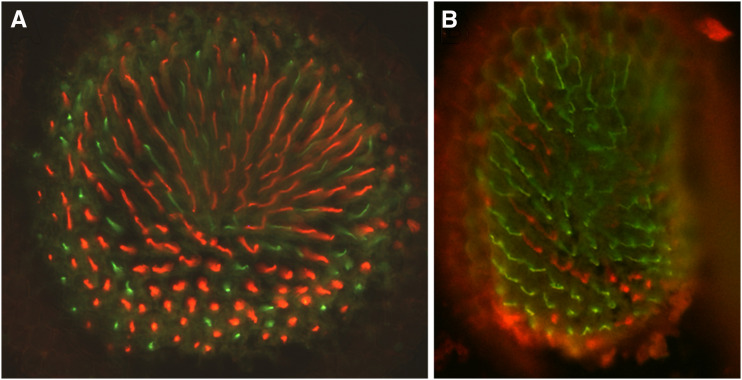
Homozygous loss of *slit* dramatically increases *Rh3*%. A) The white-eyed, but otherwise wild-type *cn* *bw* retina with R7 cells stained for *Rh3* (green) or *Rh4* (red). This eye section has 31% *Rh3* (*n* = 232). B) Flies homozygous for the *slit*^*EP937*^ insertion (identified in our screen) have 60.4% *Rh3* (*n* = 618).

As an additional step in validating the effects of mutations on *Rh3*%, we examined a subset of insertion strains as homozygotes. The rationale for the heterozygous LOF (HetLOF) screen was based upon the assumption that reduction in the level of a rate-limiting regulator of R7 photoreceptor differentiation (even in heterozygotes) would demonstrate a change in *Rh3*%. If this was true, then our expectation is that animals homozygous for the insertion (Homozygous Loss of Function, HomLOF) would have a further reduction in that critical regulator and would demonstrate a larger change in *Rh3*%. Figure 4 shows that for the viable homozygotes tested, the change in *Rh3*% is more extreme than in either the LOF (HetLOF) or GOF screens in most cases, as shown for the dramatic increase of *Rh3*% in *slit*^*EP937*^ (*sli*) mutants compared to *cn* *bw* controls (Figure 4 and Figure 5).

## Discussion

The genetic screens described here were designed to identify transposon insertion strains that showed alterations in the stochastic differentiation of R7 photoreceptor cells expressing *Rh3* or *Rh4*. Potential alterations were defined as significant *Rh3*% change in the GOF or LOF screens. The screens identified 69 unique candidate genes. Many of the genes identified in the screen have previously identified protein-protein and genetic interaction between themselves and the known regulators of R7 cell differentiation, *klu*, *tgo* and *ss*. Preliminary validation of candidates by examination of homozygous insertions (HomLOF) showed substantially increased effects on *Rh3*%.

Despite the intriguing results of the screens, a series of caveats related to the use of the *P{EP}* insertions lines should be noted. *P*-elements insert in a biased manner within genomic hot-spots, preferentially into gene promoters and replication origins (Spradling *et al.* 2011). Therefore, insertions do not represent a random sample of the genome and are biased with respect to the regions targeted of an endogenous gene. The GOF screen is predicated on the assumption that the *P{EP}* element is inserted in a region 5′ of the transcription initiation site of the endogenous gene, as shown in Figure 1. Ideally, the cross of *P{EP}* and *P{sevEP-GAL4}* strains would produce offspring expressing the full-length protein of the encoded gene in the pattern of *P{sevEP-GAL4}*. This is not necessarily the case because of the orientation and position of the *P{EP}* insertion. As indicated in Table 1, several of the *P{EP}* lines carry insertions that are in the opposite orientation of the transcribed gene (reverse EP orientation), *Fur2*^*EP1493*^, *Gβ13F^EP1071^*, *RhoGAP18^EP1326^* and *GIIIspla2*^*EP1516*^. For these insertion lines and for similarly oriented strains within the screen, statistically significant GOF effects may not be due to expression of the indicated gene, and the lack of a significant effect for a particular *P{EP}* line is not a sufficient test of the indicated gene. Furthermore, the position of the *P{EP}* insertion in an intron or within the gene is also not a test of expression of the full-length gene product, as in the case of *Imp*^*EP760*^. These caveats also apply to a portion of the 29 additional insertion lines showing a statistically significant change in *Rh3*% in the GOF screen that are indicated in Supplemental Table S2.

The *P{EP}* strain library was generated by *P*-element transposition, which has been used as a method of mutagenesis for many years (Rubin *et al.* 1982). Each *P{EP}* strain is associated with a specific genomic location, based on sequenced flanking regions, and annotated within Flybase as an allele of one or more genes. The heterozygous LOF screen is predicated on the assumption that insertion of the *P{EP}* element may cause loss of function of the annotated gene. This too is not necessarily the case and is also dependent on the specific insertion site. Associated caveats include: 1) many of the *P{EP}* lines are alleles of essential genes, but are homozygous viable, suggesting that they have a partial loss of function (hypomorphs), 2) the sequenced insertion sites may be in regions of the gene that do not affect gene function in any way, 3) the insertion site may reside between two genes and affect the function of both, one, or neither gene as in the case of *GIIIspla2*^*EP1516*^, *Traf6*^*EP1516*^, 3) the observed phenotypes may be independent of the *P{EP}* insertion. This could occur because of additional mutations in the backgrounds of the strains that were used for *P{EP}* mobilization or that resulted from the mobilization event itself. In addition, male recombination (Hiraizumi 1971), or other mutations or chromosome aberrations could occur that are unlinked to the *P{EP}* insertion site (Hiraizumi *et al.* 1973; Simmons and Lim 1980). Also, the 411 insertion strains that lack a phenotype in the GOF, LOF screens or the Diff comparison do not serve to exclude those nearby genes from involvement in the regulation of *Rh3* expression. Rather the function of the study was simply to identify those insertions strains that do show a phenotype.

Despite these caveats, the current study contributes significantly to our understanding of the specification of R7 photoreceptor cells and the regulation of *Rh3* and *Rh4* expression. Many of the genes identified in the screen have previously identified protein-protein and genetic interaction between themselves and the known regulators of R7 cell differentiation, *klu*, *tgo* and *ss*. Preliminary validation of candidates by examination of homozygous insertions (HomLOF) showed substantially increased effects on *Rh3*%. The candidate genes identified encode proteins comprising a broad range of biological functions. Of the selected genes highlighted in Table 1, *SmD2* encodes an RNA binding protein that is involved in pre-mRNA splicing (Mount and Salz 2000). *Tpst* shares homology with tyrosyl-protein sulfotransferases (Gaudet *et al.* 2011), localizes to the Golgi apparatus and is involved in protein secretion (Bard *et al.* 2006). *Fur2* encodes a proprotein convertase that mediates ligand activation through protein cleavage (Kunnapuu *et al.* 2009). *Gβ13F* encodes a G-protein β-subunit that mediates G-protein coupled receptor signaling and modulates *hedgehog* signaling (Li *et al.* 2018). *grau* encodes a zinc-finger transcription factor (Chen *et al.* 2000). *Imp* encodes an mRNA binding protein that promotes and regulates transcript targeting, and plays a role in axonal remodeling, synaptogenesis and oogenesis (Boylan *et al.* 2008; Medioni *et al.* 2014). *Arp3* encodes an actin related protein that is required for myoblast fusion and axonal arborization and synapse formation (Richardson *et al.* 2007; Koch *et al.* 2014). *RhoGAP18B* encodes a GTPase activating protein that plays a role in the behavioral response to ethanol and neuromuscular junction formation (Laviolette *et al.* 2005; Rothenfluh *et al.* 2006). *Tao* encodes a mitogen-activated protein kinase kinase kinase and acts together with *hippo* (*hpo**)* to activate *warts** (**wts**)*-mediated repression of *yorkie** (**yki**)* (Poon *et al.* 2011). *GIIIspla2* encodes a phospholipase A2 that interacts genetically with the E3 ubiquitin ligase *parkin* (Cha *et al.* 2005).

The major findings of the current work are: 1) the results provide strong support for the hypothesis that *Rh3*% is sensitive to gene dosage effects through loss of function or gain of function as might be expected for a stochastic, cell-autonomous biological process, 2) numerous genes in addition to *klu*, *tgo* and *ss*, are likely to play a role in regulating this process, 3) the identification of cell-cell signaling and cell surface molecules suggests that R7 cell subtype specification may not be exclusively cell-autonomous, but likely involves inputs from other cell types or tissues. Interestingly, the interaction network defined by the candidate genes contains several genes previously shown to influence specification of *Rh3* or *Rh4* expression in R7 photoreceptor cells (*Rh3*%) as well as the inductive signal that is thought to coordinate the expression of opsin genes in adjacent R7 and R8 photoreceptor cells within individual ommatidia. These include (*Egfr*) (Birkholz *et al.* 2009a) that is required in both processes, as well as *Merlin* (*Mer*), *wts*, *hpo*, *kibra*, *yki* (Jukam and Desplan 2011; Jukam *et al.* 2013) and *thickveins* (*tkv*) (Wells *et al.* 2017) that coordinate opsin gene expression in R7 and R8 cells. Furthermore, one of the candidate genes identified in the screen, *Fur2*, has also been shown to regulate the induction of *Rh5* expression in R8 cells (Wells *et al.* 2017), although this could be secondary to the effect on *Rh3* expression in R7 cells shown in the current study. These results suggest numerous mechanisms through which processes in the R7 cell responsible for R7y *vs.* R7p cell fate may couple to inductive signaling that specifies paired R8y and R8p cell fates, respectively. Further validation of candidate genes using precise excision to test phenotypic reversion, additional mutant alleles, and RNAi will provide definitive evidence of their involvement. The initial analyses here are insufficient to determine the precise mechanism of action of the identified candidate genes. They could potentially function cell autonomously within the R7 photoreceptor cell or play roles outside of the R7 cell. The candidate genes may interact genetically with, or be independent of, *ss*. Finally, the genes identified in this study may play an instructive or a permissive role in R7 cell differentiation. Additional experiments will be required to further validate the candidate genes and resolve these mechanistic questions.
